# 32. Host Immune-Protein Signature Combining TRAIL, IP-10 and CRP for Early and Accurate Prediction of Severe COVID-19 Outcome

**DOI:** 10.1093/ofid/ofab466.032

**Published:** 2021-12-04

**Authors:** Alon Angel, Niv Samuel Mastboim, Oded Shaham, Tahel Ilan Ber, Roy Navon, Einav Simon, Michal Rosenberg, Yael Israeli, Mary Hainrichson, Noa Avni, Eran Reiner, Paul Feigin, Kfir Oved, Boaz Tadmor, Pierre Singer, Ilya Kagan, Shaul Lev, Dror Diker, Amir Jarjou’i, Ramzi Kurd, Eli Ben-Chetrit, Guy Danziger, Cihan Papan, Sergey Motov, Maanit Shapira, Michal Stein, Adi Klein, Tanya Gottlieb, Eran Eden

**Affiliations:** 1 MeMed, Haifa, Israel, Haifa, Hefa, Israel; 2 MeMed Diagnostics, Tirat Carmel, HaZafon, Israel; 3 MeMed, Haifa, HaZafon, Israel; 4 Technion-Israel Institute of Technology, Haifa, HaZafon, Israel; 5 Rabin Medical Center, Hasharon, Petach Tikva, Israel, Tel Aviv University, Israel, Petach Tikva, HaMerkaz, Israel; 6 Rabin Medical Center, Petah Tikva, HaMerkaz, Israel; 7 Hasharon Hospital-Rabin Medical Center, Petah Tikva, HaMerkaz, Israel; 8 Shaare Zedek Medical Center, Jerusalem, Yerushalayim, Israel; 9 Saarland University Hospital, Homburg, Saarland, Germany; 10 Maimonides Medical Center, New York, New York; 11 Hillel Yaffe Medical Center, Hadera, HaZafon, Israel

## Abstract

**Background:**

Accurately identifying COVID-19 patients at-risk to deteriorate remains challenging. Dysregulated immune responses impact disease progression and development of life-threatening complications. Tools integrating host immune-protein expression have proven useful in determining infection etiology and hold potential for prognosticating disease severity.

**Methods:**

Adults with COVID-19 were enrolled at medical centers in Israel, Germany, and the United States (Figure 1). Severe outcome was defined as intensive care unit admission, non-invasive or invasive ventilation, or death. Tumor necrosis factor related apoptosis inducing ligand (TRAIL), interferon gamma inducible protein-10 (IP-10) and C-reactive protein (CRP) were measured using an analyzer providing values within 15 minutes (MeMed Key®). A signature indicating the likelihood of severe outcome was derived generating a score (0-100).

Description of derivation cohort

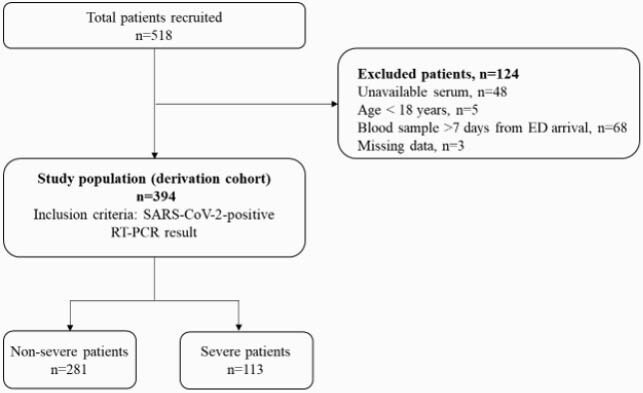

RT-PCR, reverse transcription polymerase chain reaction.

**Results:**

Between March and November 2020, 518 COVID-19 patients were enrolled, of whom 394 were eligible, 29% meeting a severe outcome. Age ranged between 19-98 (median 61.5), with 59.1% male. Patients meeting severe outcomes exhibited higher levels of CRP and IP-10 and lower levels of TRAIL (Figure 2; p < 0.001). Likelihood of severe outcome increased significantly (p < 0.001) with higher scores. The signature’s area under the receiver operating characteristic curve (AUC) was 0.86 (95% confidence interval: 0.81-0.91). Performance was not confounded by age, sex, or comorbidities and was superior to IL-6 (AUC 0.77; p = 0.033) and CRP (AUC 0.78; p < 0.001). Clinical deterioration proximal to blood draw was associated with higher signature score. Scores of patients meeting a first outcome over 3 days after blood draw were significantly (p < 0.001) higher than scores of non-severe patients (Figure 3). Moreover, the signature differentiated patients who further deteriorated after meeting a severe outcome from those who improved (p = 0.004) and projected 14-day survival probabilities (p < 0.001; Figure 4).

TRAIL, IP-10, CRP and the severity signature score are differentially expressed in severe and non-severe COVID-19 infection

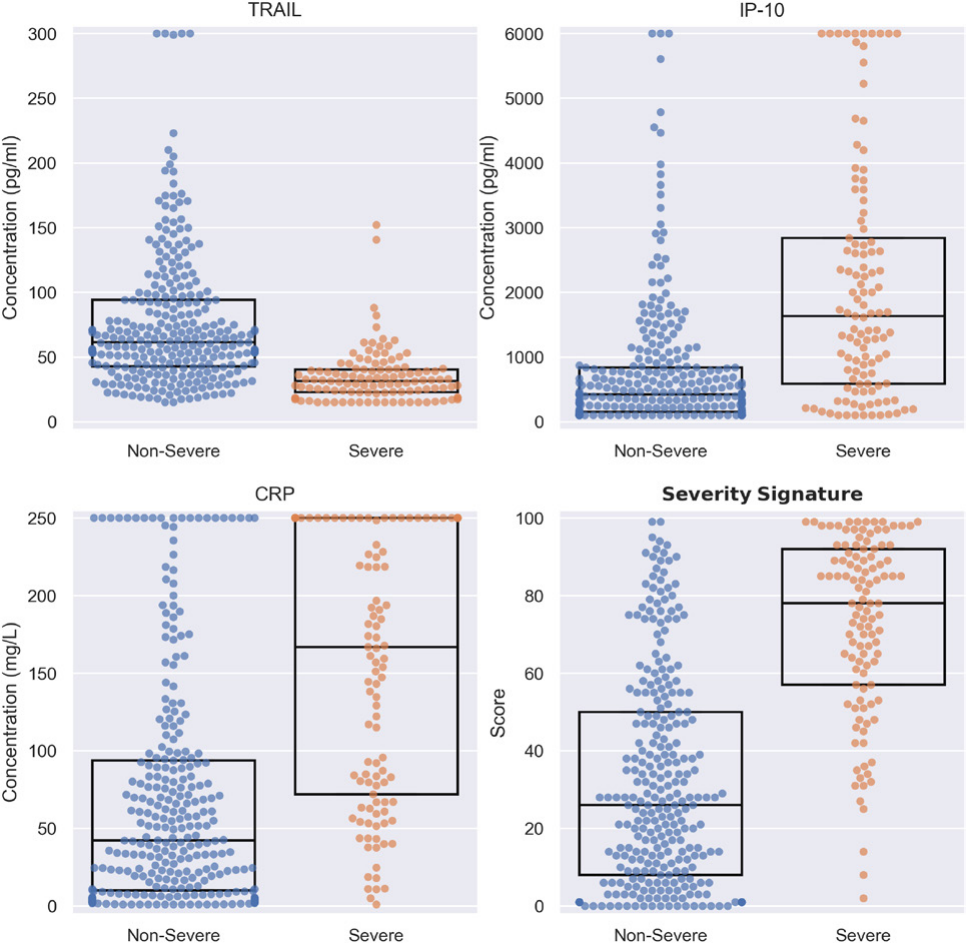

Dots represent patients and boxes denote median and interquartile range (IQR)

The signature score of patients meeting a severe outcome on or after the day of blood draw is significantly (p < 0.001) higher than the signature score of non-severe patients.

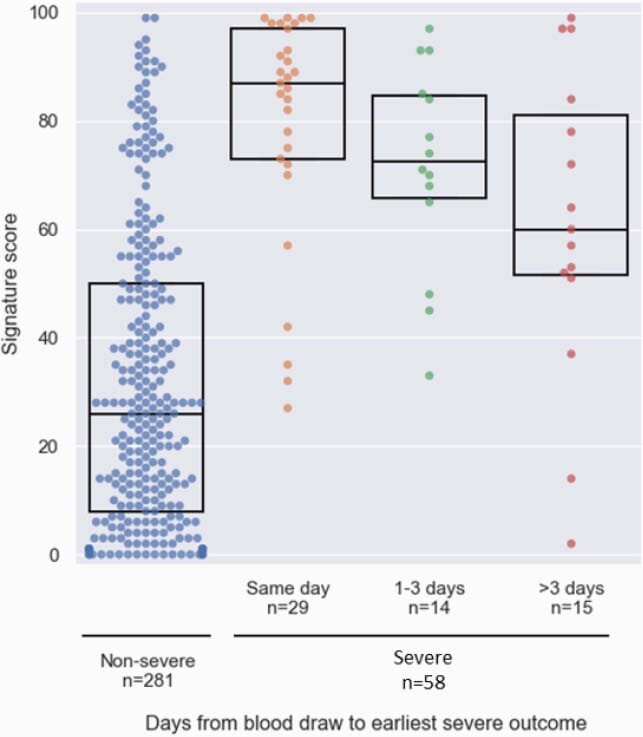

Dots represents patients and boxes denote median and IQR

Kaplan-Meier survival estimates for signature score bins

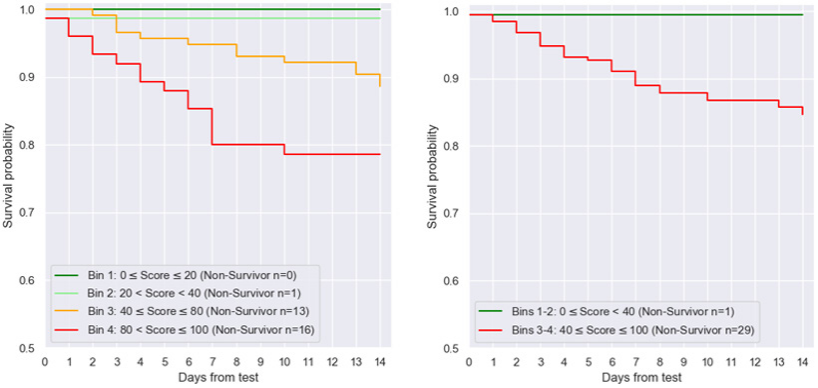

**Conclusion:**

The derived signature combined with a rapid measurement platform has potential to serve as an accurate predictive tool for early detection of COVID-19 patients at risk for severe outcome, facilitating timely care escalation and de-escalation and appropriate resource allocation.

**Disclosures:**

**Alon Angel, n/a**, **MeMed** (Employee, Shareholder) **Niv Samuel Mastboim, BSc**, **MeMed** (Employee, Shareholder) **Oded Shaham, PhD**, **MeMed** (Employee, Shareholder) **Tahel Ilan Ber, MD**, **MeMed** (Employee, Shareholder) **Roy Navon, MSc**, **MeMed** (Employee, Shareholder) **Einav Simon, PhD**, **MeMed** (Employee, Shareholder) **Michal Rosenberg, PhD**, **MeMed** (Employee) **Yael Israeli, PhD**, **MeMed** (Employee) **Mary Hainrichson, PhD**, **MeMed** (Employee, Shareholder) **Noa Avni, PhD**, **MeMed** (Employee) **Eran Reiner, MD**, **MeMed** (Employee) **Kfir Oved, MD, PhD**, **MeMed** (Board Member, Employee, Shareholder) **Ilya Kagan, MD**, **MeMed** (Scientific Research Study Investigator) **Shaul Lev, M.D**, **MeMed** (Scientific Research Study Investigator) **Dror Diker, MD**, **MeMed** (Scientific Research Study Investigator) **Amir Jarjou’i, MD**, **MeMed** (Scientific Research Study Investigator) **Ramzi Kurd, MD**, **MeMed** (Scientific Research Study Investigator) **Guy Danziger, MD**, **MeMed** (Scientific Research Study Investigator) **Cihan Papan, MD**, **MeMed** (Scientific Research Study Investigator) **Sergey Motov, MD**, **MeMed** (Scientific Research Study Investigator) **Maanit Shapira, Ph.D**, **MeMed** (Scientific Research Study Investigator) **Tanya Gottlieb, PhD**, **MeMed** (Employee, Shareholder) **Eran Eden, PhD**, **MeMed** (Board Member, Employee, Shareholder)

